# Asymmetric Correlation between Experienced Parental Attachment and Event-Related Potentials Evoked in Response to Parental Faces

**DOI:** 10.1371/journal.pone.0068795

**Published:** 2013-07-02

**Authors:** Junqiang Dai, Hongchang Zhai, Anbang Zhou, Yongyuan Gong, Lin Luo

**Affiliations:** Department of Psychology, Educational College of Guangzhou University, Guangzhou, China; Charité University Medicine Berlin, Germany

## Abstract

This study aims to explore the modulation effects of attachment relationships with parents on the neural correlates that are associated with parental faces. The event-related potentials elicited in 31 college students while viewing facial stimuli of their parents in two single oddball paradigms (father vs. unfamiliar male and mother vs. unfamiliar female) were measured. We found that enhanced P3a and P3b and attenuated N2b were elicited by parental faces; however, the N170 component failed to discriminate parental faces from unfamiliar faces. An experienced attachment relationship with the father was positively correlated to the P3a response associated with the father’s face, whereas no correlation was found in the case of mothers. Further exploration in dipole source localization showed that, within the time window of the P300, distinctive brain regions were involved in the processing of parental faces; the father’s face was located in the medial frontal gyrus, which might be involved in self effect, and the anterior cingulate gyrus was activated in response to the mother’s face. This research is the first to demonstrate that neural mechanisms involved with parents can be modulated differentially by the qualities of the attachments to the parents. In addition, parental faces share a highly similar temporal pattern, but the origins of these neural responses are distinct, which could merit further investigation.

## Introduction

Evolutionarily speaking, parents are the most salient stimulus and mark the apex of biological significance; the attachment bonding of parent–child plays an important role in promoting humans’ survival and healthy development [1]. Delineating the neural mechanisms that underlie the processing of parental faces could result in a better understanding of the nature of the parent–child bond; this goal has led to a rise in brain imaging and electrophysiological studies that reported differences in neural responses when individuals process the faces of their parents compared to others. These studies include fMRI studies that report increased activity in areas of the bilateral cingulate gyrus and the right superior frontal gyrus when viewing parental faces compared to unfamiliar faces, and this activation did not correlate with age, spatial distance or the time spent with their parents [2]. While the PCC-Pcu (posterior cingulated cortex-precuneus) showed a graded activation for one’s mother, friends and strangers, the VMPFC-ACC (ventromedial prefrontal cortex-anterior cingulate cortex) activity distinguished the specific processing of the mother from a close friend [3]. In addition, research reported that mothers’ faces elicit more activity in core and extended brain regions compared to fathers’ faces, which suggests that mothers’ faces can be more salient than fathers’ faces [4]. On the other hand, event-related potential (ERP) studies found that enhanced P3 or LPP responses were elicited when individuals process personally significant faces relative to unknown or less significant faces [5–9], which demonstrates that in the late stage of information processing, the neural mechanism was sensitive to the motivational significance that underlies personal salient faces.

Recently, it was suggested that human brain activities that are associated with social cognition might correlate with the measures of relationship qualities or characteristics. For example, brain electrophysiology in response to parental faces positively correlates with perceived parental support [6]; those participants who perceived their relationship with parents to be more supportive exhibited a greater LPP for their parent’s face, whereas a negative interaction attenuated this association. With regard to the perspective of the mother, the stronger the parent–child relationship that is perceived by the mother, the larger the P3 and LPP responses associated with an infant’s face that were elicited [9]. The methodology of integrating the neural response and relationship quality could facilitate our exploration toward delineating the hierarchical structure of social networks [6] and provide a more detailed explanation for the neural mechanisms that are associated with social stimuli.

For humans, parents were the most direct attachment-related objective; infancy in humans involves psychological attachment to parents, and the attachment is usually stable during adulthood [10]. According to the attachment theory, attachment accomplishes basic regulatory functions and includes excitatory and inhibitory neural circuits by which attachment affects the appraisal of an object [11]. Some evidence has indicated that attachment affects the cognitive accessibility to social stimuli; for example, those individuals with avoidant attachment orientation have a specific capacity to differentiate threat from non-threat stimuli at an early stage (50-80 ms post-stimulus) of information processing [12]; regulatory emotional capacities of the vasopressin and oxytocin neuropeptide systems would also be influenced by the attachment experience [13]. Currently, no study to our knowledge has addressed the modulation effect of the attachment with parents on the neural correlates of the parents, and considering that previous studies did not measure separately the relationship qualities with the parent, we can extend the previous research and examine whether paternal or maternal attachment relationships would modulate the neural patterns in response to either the father’s face or the mother’s face or both. Furthermore, whether any difference exists in the neuropsychological response to the father’s and mother’s face also merits further exploration.

Measuring ERPs provides data with excellent temporal resolution, which reflects different stages of processing and indexing with distinct cognitive mechanisms, such as the facial perceptual component, the N170, an occipito-temporal negative amplitude that peaks at a latency of approximately 170 ms post-stimulus, which is associated with the structural encoding of faces [14]. With regard to this component, it was still controversial as to whether it can reflect face familiarity or not; some researchers found familiarity effects in the N170 [15,16], but other researchers revealed the opposite finding [9,17,18]. Recent research on other early electrophysiological potentials has primarily focused on the N2b component; a review study of the N2b suggested that this component might distribute across the whole brain during different task paradigms or stimulus modalities. Specifically, the anterior N2b might reflect aspects of cognitive control, while the posterior N2b might reflect visual attention [19,20]. Another ERP component applied broadly in face processing research is the P300, which is a family of distinct late positive components that have divergent distributions across the entire brain, reflecting distinct mental operations [21,22]. Because the P300 potential would be enhanced after the target was determined to be different from the standard stimulus, if a low frequency of significant faces were interspersed amidst a higher frequency of non-significant faces, the P300 response could be used to evaluate the ability to distinguish significant faces from other face types. One of the subcomponents, parietal P3b, was considered to be generated from temporal-parietal activity associated with voluntary attention and subsequent working memory processing [21,23,24], which was often obtained with the oddball paradigm, in which two types of stimuli are presented in a random series; one stimulus appears significantly less frequently, and the subjects were instructed to respond to the infrequent event. This component would be determined by the targetness [25], visual familiarity [5] or working memory [24]. However, another subcomponent, frontally distributed P3a, has been shown to be more sensitive to those stimuli that contain a special nature with regard to humans. For example, in the study of Bobes et al. (2007), faces of close relatives or friends (which contain social significance) and artificially-learned faces (which are devoid of social significance) were both used as infrequent targets in two oddball series; only acquaintance faces elicited an additional frontally distributed P3a component, which implies that the emotional significance is a necessary condition for its generation. Furthermore, as compared to romantically uninvolved singles, both parents and lovers also exhibited a P3a response to an infant face [26]. To date, little research has addressed the effect of parental biological significance on the P3a component.

In light of the above, we aimed to use the P3a (but not exclude the N170, N2b and P3b) as an objective measure in exploring how the parental attachment relationship modulates ERP patterns in response to the father’s and mother’s face, while separately measuring the electrophysiological response and attachment qualities with the parents. To augment the ERP components mentioned above, parental faces were embedded in the oddball series containing unfamiliar faces as the frequent stimulus. In total, 31 Chinese undergraduate college students rated their attachment qualities to their father and mother in three dimensions, including trust, communication and alienation, and then engaged in two oddball experiments (father vs. a male stranger face and mother vs. a female stranger face). The participants were instructed to press the button when their parents’ faces were presented.

Several hypotheses were proposed. First, the P3a component was sensitive to the stimulus with biological significance (i.e., parental faces), and it can differentiate parental faces from unfamiliar faces. With regard to the parietally distributed P3b, parental faces were hypothesized to elicit a larger response because of the cognitive task of the experiment and the attentive recognition of parental faces. While N2b can reflect the early processing of a face, we were eager to find an early difference between the faces of parents and unfamiliar individuals, which is in line with previous studies. In addition, considering the inconsistent findings on the familiarity effect of the N170, we made no prediction for the N170 component. Second, because the P3a was found to be related to personally salient significance, a high quality attachment with parents could promote the P3a response to parents, as shown in one previous study. Finally, within the time window of the P300 response, the dipole source localization of parental faces could be located in differential brain regions, based on the idea that different types of face-derived information are processed in distinct and specialized brain sub-systems, as proposed by [27].

## Methods

The Academic Ethics Committee of Guangzhou University approved the study and all participants and their parents provided written informed consent. The parents whose photographs are presented here have given written informed consent, as outlined in the PLOS consent form, permitting us to publish, reuse and reprint their photographs.

### Participants

The sample included 31 Chinese undergraduate college students (15 male and 16 female) who were enrolled in different majors and who received monetary rewards for participating in the study. Participants were between the ages of 19 and 23 years old (*M* = 20.68, *SD* = 1.40). All of the participants were right-handed with normal vision, free of psychological disorders and not currently under the influence of drugs, alcohol or psychotropic medications. All of the participants signed the informed consent before the experiment.

### Stimuli and measures

#### Facial stimulus

**Figure 1:**
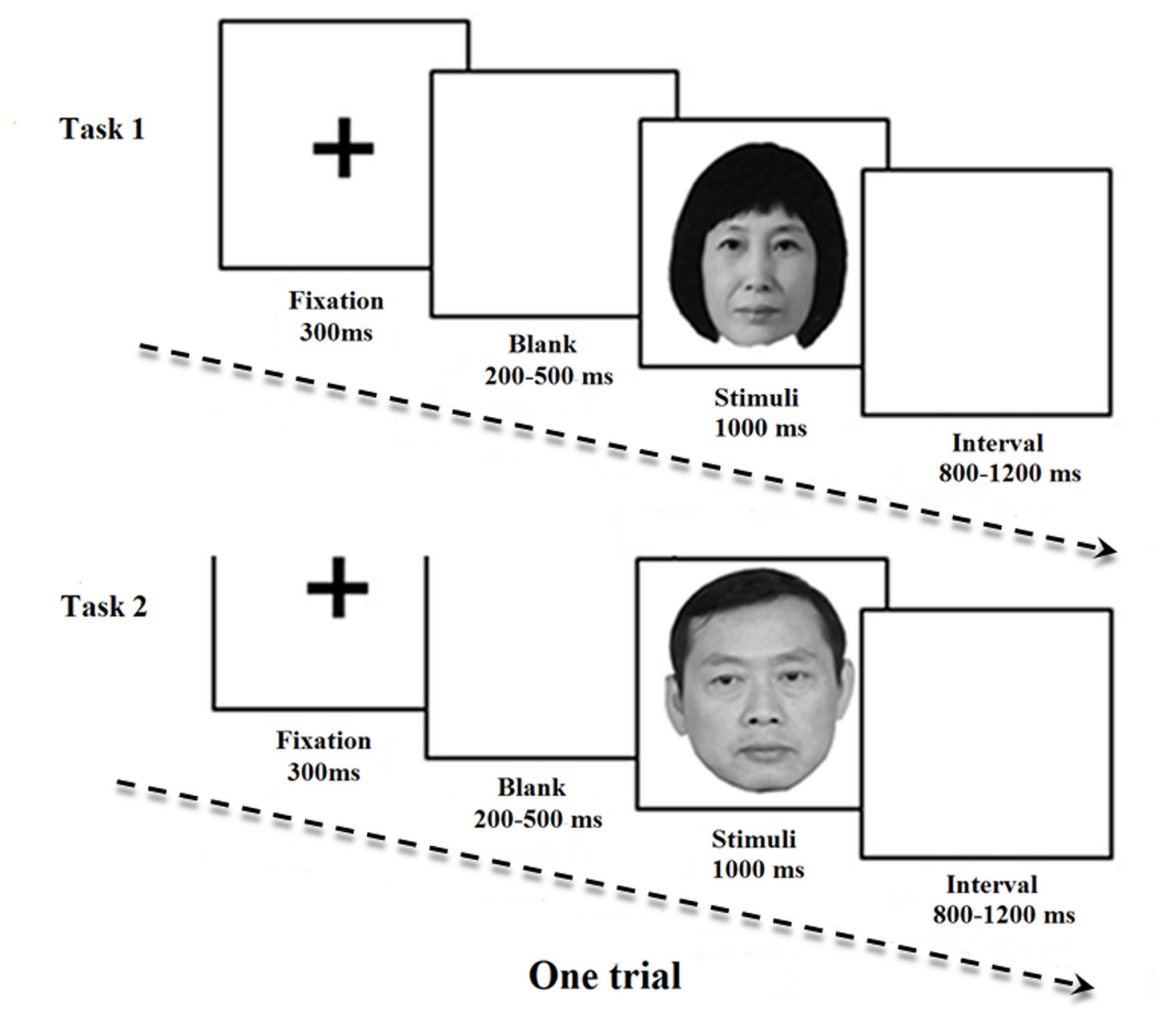
Recording procedure and an example of the facial stimuli. In each trial, the participants were asked to respond as soon as their parents’ faces were presented and to ignore male/female strangers’ faces. All of the participants were asked to finish two single oddball tasks, and the task order was balanced across the participants. The parents whose photographs are presented here have given written informed consent, as outlined in the PLOS consent form, permitting us to publish, reuse and reprint their photographs.

Color photographs of the parents were taken from the neck up, against a light gray background. All of the parents assumed a neutral expression for the photograph and wore a gray scarf to obscure their neckline and clothing; earrings and other jewelry were removed. To avoid the perceptual information that is generated by skin color, which could affect the participants’ behavioral and neurophysiological responses, the pictures presented on-screen were in grayscale (see Figure 1). Facial stimuli were loaded into Adobe Photoshop CS Version 5.0 to appropriately control the size, color and brightness.

The sample tested in the current study came from university faculty, and all of the parents were from the Han nationality of China; some participants’ parents were used as unfamiliar faces for other participants. The pictures of the parents and unfamiliar faces were matched according to age, and all of the participants were asked to ensure whether they had any impression of the unfamiliar faces before the experiment.

#### The Inventory of Parent and Peer Attachment (IPPA)

The IPPA [28] contains three dimensions (trust, communication and alienation) and is derived from three items that assess trust (e.g., “My father/mother respects my feelings.”), three items that assess communication (e.g., “If my father/mother knows something is bothering me, he/she asks me about it.”) and four items that assess alienation (e.g., “Talking over my problems with my father/mother makes me feel ashamed or foolish.”). The items are rated on five-point Likert-type scales that range from 1 (little or none) to 5 (the most). The IPPA has demonstrated good psychometric properties in previous studies, and the internal consistency (Cronbach’s alpha) in the current sample was 0.780 for maternal attachment and 0.778 for paternal attachment.

### Procedure

Two single classical oddball paradigms (father vs. male strangers and mother vs. female strangers) were used. In each paradigm, the photographs of the father/mother served as targets, whereas the photographs of the unfamiliar male/female served as non-targets; the order of the paradigms was balanced across the participants. The participants were instructed to acknowledge their recognition of a target by pressing either the F or J button placed under their left and right index fingers, respectively, while ignoring unknown stimuli; the buttons pressed were also balanced across the participants and the parents’ faces.

The participants sat in front of a table that was approximately 75 cm from the video monitor that delivered the stimuli, with a vertical visual angle of 5.3° and a horizontal visual angle of 3.6°. Each oddball experiment comprised 240 trials that were separated into 4 blocks of 60 trials each. The stimulus order was randomized in each block, with the photographs of the father’s/mother’s faces (target) presented 12 times (20%) within each block and photographs of the unfamiliar male’s/female’s faces (non-target) presented 48 times (80%) per block, with each of the six photographs of the unfamiliar faces appearing 8 times per block. The trial sequence was a black fixation cross presented for 300 ms on a white background, with the white background varied randomly between 200–500 ms. A facial stimuli was presented for 1000 ms or until a response was made, and there was an inter-stimulus interval (ISI) that varied randomly between 800 and 1200 ms (Figure 1).

#### ERP recording and data reduction

Electroencephalographic activity was recorded using a 64-channel BrainAmp amplifier (BrainProducts, Germany) with a 64-electrode Braincap. The electrodes were placed according to the 10–20 System and were referenced during the recording to an additional reference electrode placed between Cz and Fz, with a forehead ground; the impedance of all of the electrodes was less than 5 kΩ. Additional electrodes were placed on the outer canthi of the two eyes and on the infra-orbital ridges of the right eye, to record the horizontal and vertical EOG (electrooculography). No filter was used during the recordings. The EEG and EOG recordings were digitized with a sampling rate of 500 Hz.

Following the recording, EEG data were band pass filtered at 0.1–30 Hz (slope 12 dB/octave) and recalculated using linked mastoids as a reference [(TP9 + TP10)/2]. The analyzed time epoch for each event was 1200 ms (200 ms pre-stimulus and 1000 ms post-stimulus). To avoid eye movement and other artifacts, all of the epochs that exceeded ±80 µV in any channel were excluded from further analyses. Following the off-line analyses, participants with fewer than 50% artifact-free trials for any face condition were removed from the sample. Finally, no participant was excluded, a grand average ERP waveform and statistical analysis was performed for all the 31 participants. For each epoch, a baseline correction for the data 200 ms prior to the stimulus was performed.

### Statistical analyses

**Figure 2:**
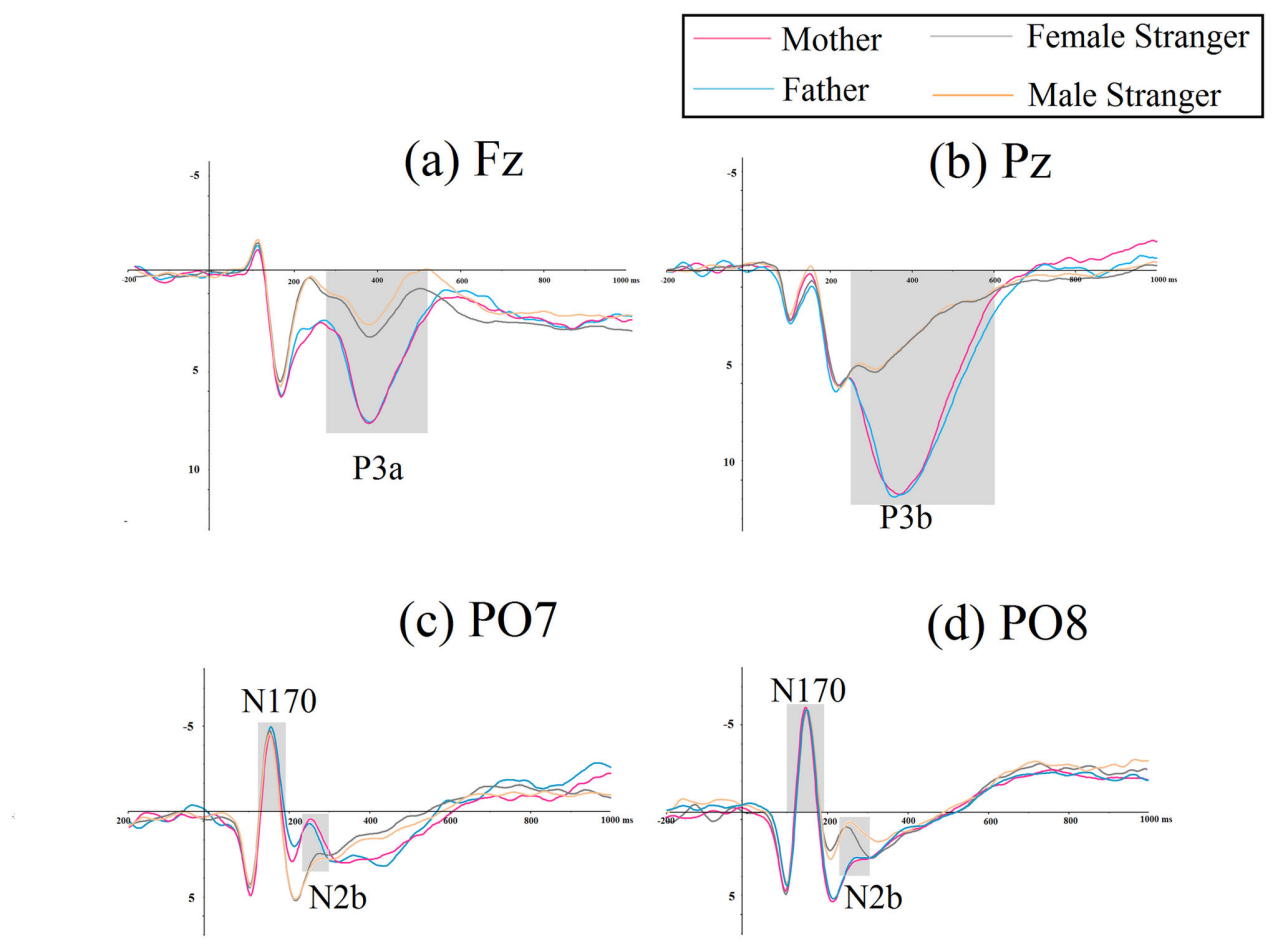
Grand average ERPs evoked by different face types. The light gray shaded areas indicate (a) a 250-550 ms time window for the P3a component at the frontal electrode, (b) a 250-650 ms time window for the P3b component at the parietal electrode, (c) and (d) a 130-180 ms time window for the N170 detection and a 200-300 ms time window for the N2b at the bilateral occipito-temporal areas.

**Figure 3:**
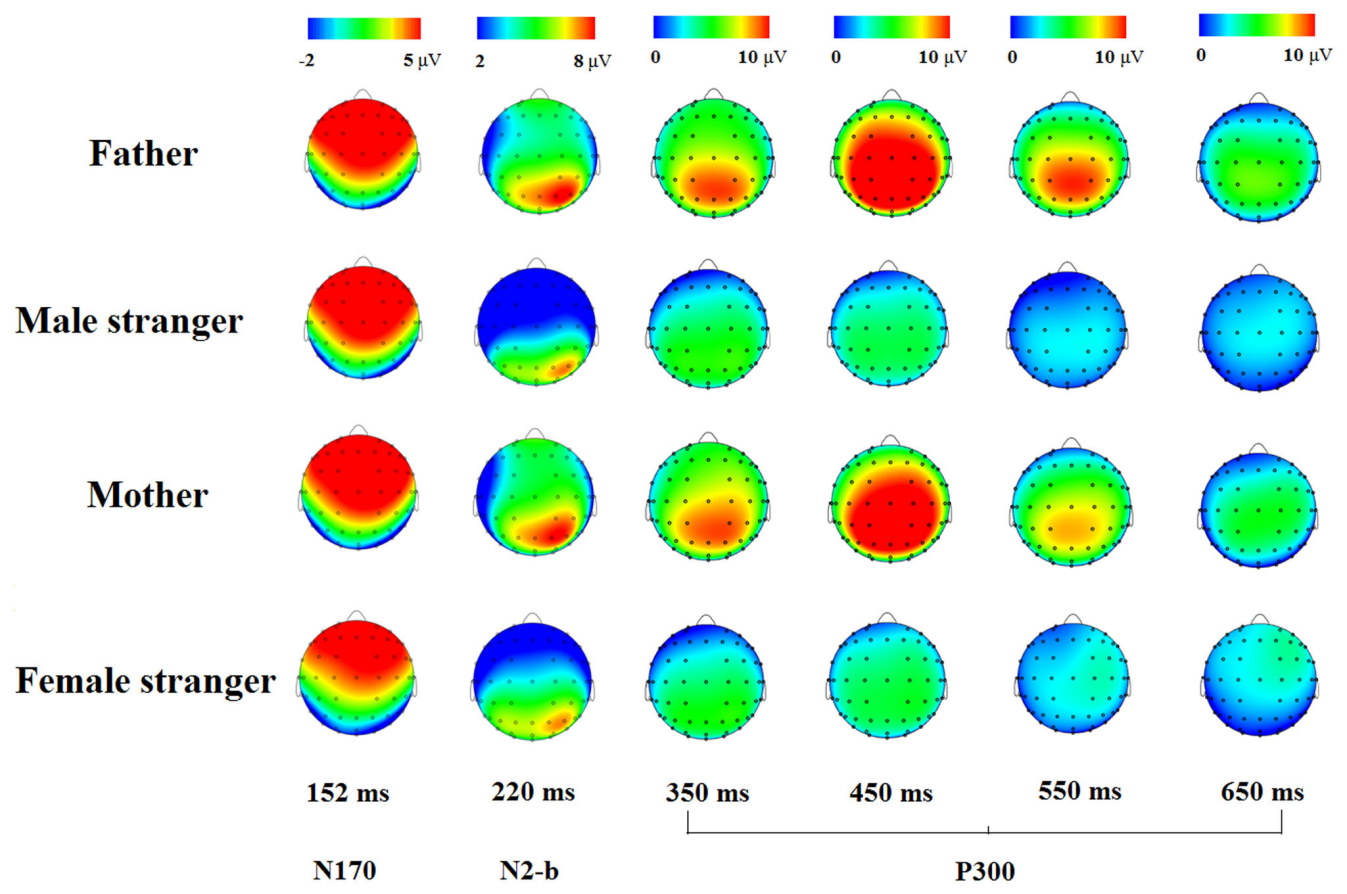
Scalp topography. Scalp topography of ERPs generated by the faces of the father, a male stranger, the mother and a female stranger, at different processing stages, as indicated by different time windows.

On the basis of the grand average waveform (Figure 2) and the topographical distribution (Figure 3), the P3a component was defined as the largest positive deflection that occurred within the time window between 250 and 550 ms at the frontal electrode sites, and the P3b component was defined as the largest positive deflection that occurred within the time windows between 250 and 650 ms at the parietal-occipital electrode sites. The mean amplitude of both P3a and P3b were measured, and the Fz, F3 and F4 electrode sites were selected for the statistical analysis of the P3a component, which applied two-way repeated measures analyses of variance (ANOVA) on the average amplitude conducted for the face category (two levels: father/mother, stranger) and electrodes (three levels: Fz, F3 and F4), with gender as a covariate ,considering that the gender affected the EPRs in previous studies [29]; the Pz, POz and Oz electrode sites were selected for the statistical analysis of the P3b component with two-way repeated measure analyses of variance (ANOVA) on the average amplitude conducted for the face (two levels: father/mother, stranger) and the electrodes (three levels: Pz, POz and Oz), also with gender as a covariate. In addition, the P3a and P3b components were compared between parental faces (father-stranger vs. mother-stranger) using the same repeated measures ANOVA. Moreover, the peak amplitude and peak latency of the face-specific components N170 and N2b over the occipito-temporal area (the PO7 and PO8 electrode sites) were also measured with a two-way repeated ANOVA. A Greenhouse-Geisser correction was applied to the *p*-values when sphericity could not be assumed.

Finally, bivariate correlation analyses were used to investigate whether neural correlates or behavioral responses to parental faces were associated with the father/mother attachment score. All of the statistical analyses were performed with SPSS 17.0 software.

### Dipole source analysis

The Brain Electrical Source Analysis program (BESA, version 5.3.7 software; MEGIS Software GmbH, Munich, Bavaria, Germany) was used to perform dipole source analyses, and the four-shell ellipsoidal head model was used. To focus on the scalp electrical activity that was related to the processing of parental faces, the averaged ERPs evoked by stranger faces were subtracted from the ERPs evoked by the paternal and maternal faces, and two difference waves were obtained. Principal component analysis (PCA) was employed in the interval of 250-650 ms for the P300 family to estimate the number of dipoles needed to explain the difference wave. When the number of dipoles was determined with PCA, the software automatically determined the dipoles’ location. The relevant residual variance criteria were used to evaluate whether this model best explained the data and accounted for the majority of the variance.

## Results

### Behavioral results

Participants were instructed to press a button when a photograph of their father/mother was presented in two different paradigms. A paired sample t-test was used to contrast the accuracy (ACC) and the reaction time (RT) in responding to a father’s and mother’s face. The results showed that the ACC was comparable for the father (*M* = 98.87, *SD* = 0.26) and mother (*M* = 99.37, *SD* = 0.19), *t*_30_ = 0.043, *p* > .05; there was also no significant difference in RT between the faces of the father (*M* = 551.36, *SD* = 18.73) and mother (*M* = 542.78, *SD* = 17.54), *t*_30_ = 0.968, *p* > .05.

Bivariate correlation analyses for the behavioral responses to father’s/mother’s faces and father/mother attachment scores showed that there was no significant correlation between the ACC and the attachment nor the RT and the attachment.

### IPPA scores

A paired sample t-test showed that there were no significant differences between the parental and maternal attachment scores (*t*_30_ = 0.402, *p* > .05; father score: 13.58±1.15; mother score: 14.10±1.09; mean with standard deviation).

### ERP data

#### Father *vs*. stranger

*P3a*: A two-way repeated-measures ANOVA with gender as a covariate revealed a main effect for the face type (*F*_1,30_ = 10.81, *p* < .005, η_p_^2^ = .61, see Figure 2a); Bonferroni-adjusted pairwise comparison showed that the father’s face elicited a larger P3a compared to the face of a male stranger at all of the frontal sites (Fz: *F*_1,30_ = 17.04, *p* < .001, η_p_^2^ = .60, 4.44±0.89µV *vs*. 0.96±0.61µV; F3: *F*_1,30_ = 7.374, *p* < .05, η_p_^2^ = .61, 4.23±0.75µV *vs*. 1.20±0.66µV; F4: *F*_1,30_ = 4.619, *p* < .05, η_p_^2^ = .53, 4.09±0.66µV *vs*. 2.05±0.62µV; mean with standard error). No significant electrode main effects or interactions were found.

*P3b*: A two-way repeated-measures ANOVA with gender as a covariate revealed the main effect for the face type (*F*_1,30_ = 19.67, *p* < .001, η_p_^2^ = .86) and the main effect for the electrode (*F*_2,60_ = 37.41, *p* < .001, η_p_^2^ = .67). A significant face and electrode interaction was also found: *F*_1,30_ = 26.47, *p* < .001, η_p_^2^ = .49; a simple effect analysis with Bonfferoni correction demonstrated that the father’s face elicited a larger P3b compared to the face of a male stranger at the Pz electrode (see Figure 2b) and the POz electrode (Pz: *F*_1,30_ = 16.95, *p* < .001, η_p_^2^ = .89, 8.57±1.12µV *vs*. 3.10±0.50µV; POz: *F*_1,30_ = 12.08, *p* < .005, η_p_^2^ = .41, 4.03±1.34µV *vs*. 1.37±0.84µV; mean with standard error).

*N2b*: A two-way repeated-measures ANOVA with gender as a covariate revealed a main effect for the face type (*F*_1,30_ = 7.462, *p* < .05, η_p_^2^ = .21, see Figure 2c & d); Bonferroni-adjusted pairwise comparison demonstrated that the father’s face elicited a smaller N2b (more positive) compared to the face of a male stranger (3.36±1.68µV *vs.* 1.97±1.32µV; mean with standard error).

#### Mother *vs*. stranger

*P3a*: A two-way repeated-measures ANOVA with gender as a covariate revealed a main effect for the face type (*F*_1,30_ = 13.04, *p* < .005, η_p_^2^ = .32, see Figure 2a); Bonferroni-adjusted pairwise comparison showed that the mother’s face elicited a larger P3a compared to the face of a female stranger at all of the frontal sites (Fz: *F*_1,30_ = 9.77, *p* < .005, η_p_^2^ = .24, 3.85±0.89µV *vs*. 1.82±0.78µV; F3: *F*_1,30_ = 12.07, *p* < .005, η_p_^2^ = .29, 3.57±0.71µV *vs*. 1.71±0.69µV; F4: *F*_1,30_ = 14.32, *p* < .005, η_p_^2^ = .36, 4.93±0.66µV *vs*. 2.67±0.75µV; mean with standard error). No significant electrode main effects or interactions were found.

*P3b*: A two-way repeated-measures ANOVA with gender as a covariate revealed a main effect for the face type (*F*_1,30_ = 12.19, *p* < .005, η_p_^2^ = .62) and a main effect for the electrode (*F*_2,60_ = 30.41, *p* < .001, η_p_^2^ = .70). A significant face and electrode interaction was also found: *F*_1,30_ = 19.87, *p* < .001, η_p_^2^ = .53; a simple effect analysis with Bonfferoni correction demonstrated that the mother’s face elicited a larger P3b compared to the face of a female stranger at the Pz electrode (see Figure 2b) and the POz electrode (Pz: *F*_1,30_ = 20.43, *p* < .001, η_p_^2^ = .84, 7.48±0.83µV *vs*. 3.05±0.63µV; POz: *F*_1,30_ = 6.90, *p* < .05, η_p_^2^ = .42, 3.54±1.31µV *vs*. 1.36±0.78µV; mean with standard error).

*N2b*: A two-way repeated-measures ANOVA with gender as a covariate revealed a main effect for the face type (*F*_1,30_ = 12.71, *p* < .005, η_p_^2^ = .31, see Figure 2c & d); Bonferroni-adjusted pairwise comparison demonstrated that the mother’s face elicited a smaller N2b (more positive) compared to the face of a female stranger (3.08±0.66µV *vs.* 1.87±0.60µV; mean with standard error).

#### Father-stranger *vs*. mother-stranger

Prior to this comparison, the artifact-free numbers of father’s and mother’s faces were analyzed using a paired sample t-test in order to ensure that these two types of stimuli were averaged with equal segments. The results of the paired sample t-test showed that there is no significant difference between the artifact-free numbers that were obtained from the father and mother paradigm (*t*_*30*_ = 1.41, *p* > .05, father face: 36.84±1.43 segments; mother face: 35.26±1.59 segments).

A two-way repeated-measures ANOVA with gender as a covariate showed that no significant main effects for the face type (father-stranger vs. mother-stranger) or the electrode or interactions were found in the analysis of the P3a or P3b amplitude.

With regard to the N170 component over bilateral occipito-temporal areas (see Figure 2c & d), the results of the current study showed that there were no significant main effects for the face types (father vs. a male stranger, mother vs. a female stranger or a comparison between the parents), the electrode or the interactions. The peak latency of N170 was approximately 152 ms over the occipito-temporal scale.

Additionally, two-way repeated-measures ANOVA with gender as a covariate also showed that there were no significant main effects for the face type (father-stranger vs. mother-stranger) or that electrode or interactions were found in the analysis of the N2b amplitude. The peak latency of N2b was approximately 220 ms over the occipito-temporal scale.

#### Bivariate correlation results

**Figure 4:**
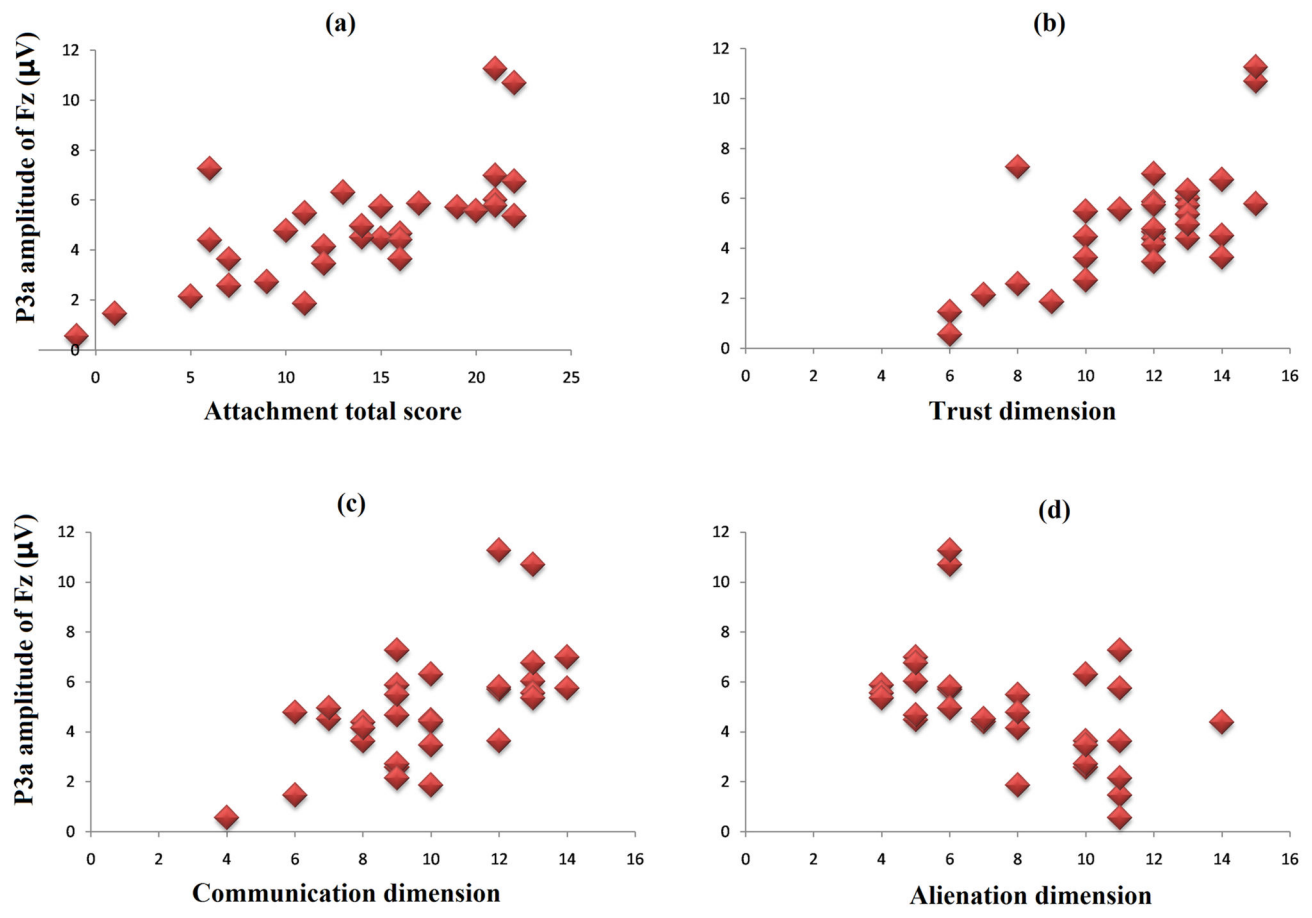
The correlations between the paternal attachment scores and P3a amplitudes evoked by a father’s face. The figure shows that the P3a amplitude was positively correlated to the total attachment score (*r* = .71, *p* < .001, Figure 4a), the trust dimension (*r* = .68, *p* < .001, Figure 4b) and the communication dimension (*r* = .60, *p* < .001, Figure 4c) and negatively correlated with the alienation dimension (*r* = -.50, *p* < .005, Figure 4d).

**Figure 5:**
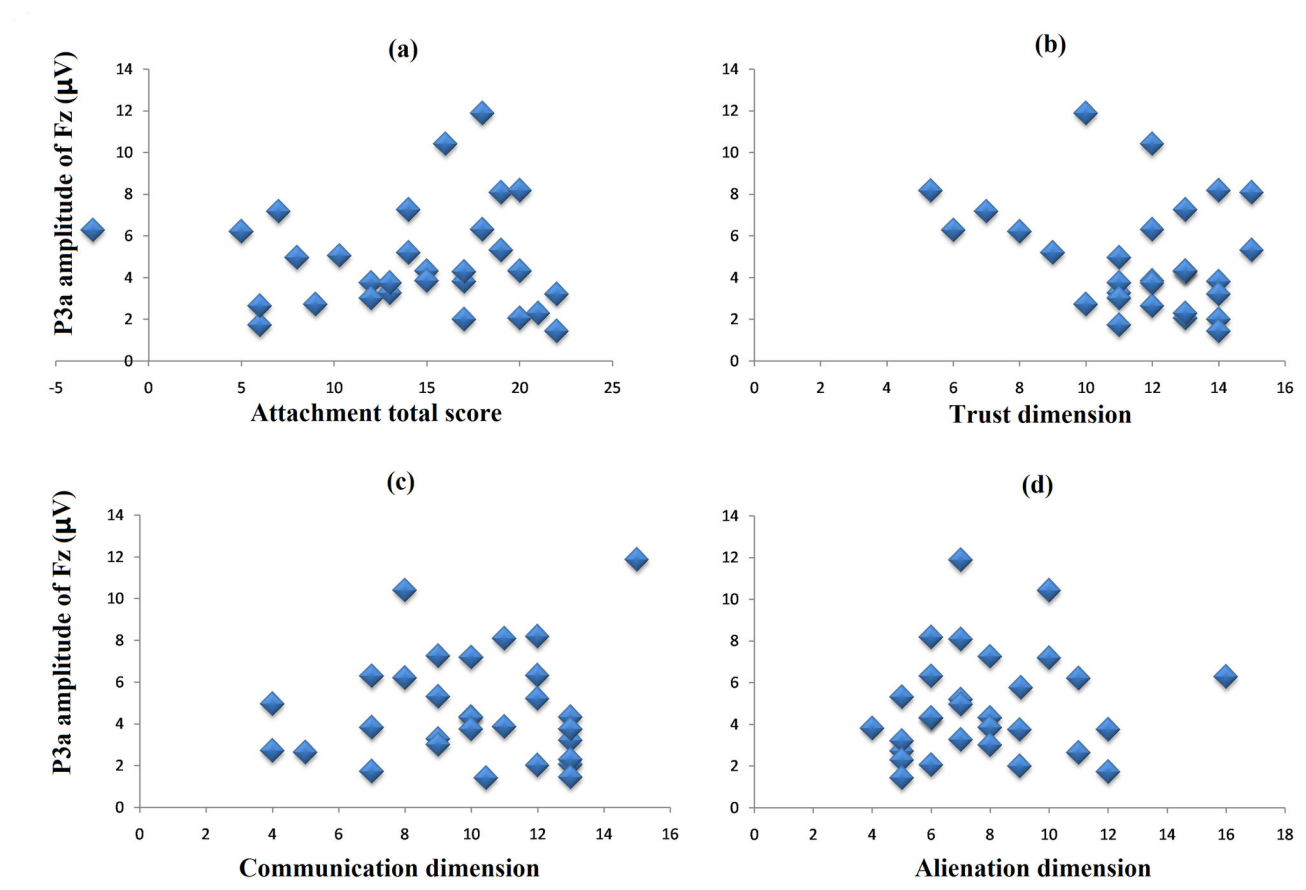
The correlations between the maternal attachment scores and the P3a amplitudes evoked by the mother’s face. Any significant correlations were found in the case of the mother (total score: *r* = .11, *p* = .302, Figure 5a; trust dimension: *r* = .10, *p* = .311, Figure 5b; communication dimension: *r* = .003, *p* = .495, Figure 5c; alienation dimension: *r* = .04, *p* = .423, Figure 5d).

The bivariate correlation analyses revealed that the P3a amplitude evoked by the fathers’ faces at the Fz electrode site was highly correlated with paternal attachment scores (total score: *r* = .71, *p* < .001, see Figure **4a**; trust dimension: *r* = .68, *p* < .001, see Figure **4b**; communication dimension: *r* = .60, *p* < .001, see Figure **4c**; alienation dimension: *r* = -.50, *p* < .005, see Figure 4d); however, the P3a evoked by the mothers’ faces had no correlation with maternal attachment scores (total score: *r* = .11, *p* > .05; trust dimension: *r* = .10, *p* > .05; communication dimension: *r* = .003, *p* > .05; alienation dimension: *r* = .04, *p* > .05), see Figure **5**.

Finally, no correlation between the P3b, N170 or N2b amplitudes evoked by the parental faces and the parental attachment scores were found.

### Source Localization

#### Father-stranger face

**Figure 6:**
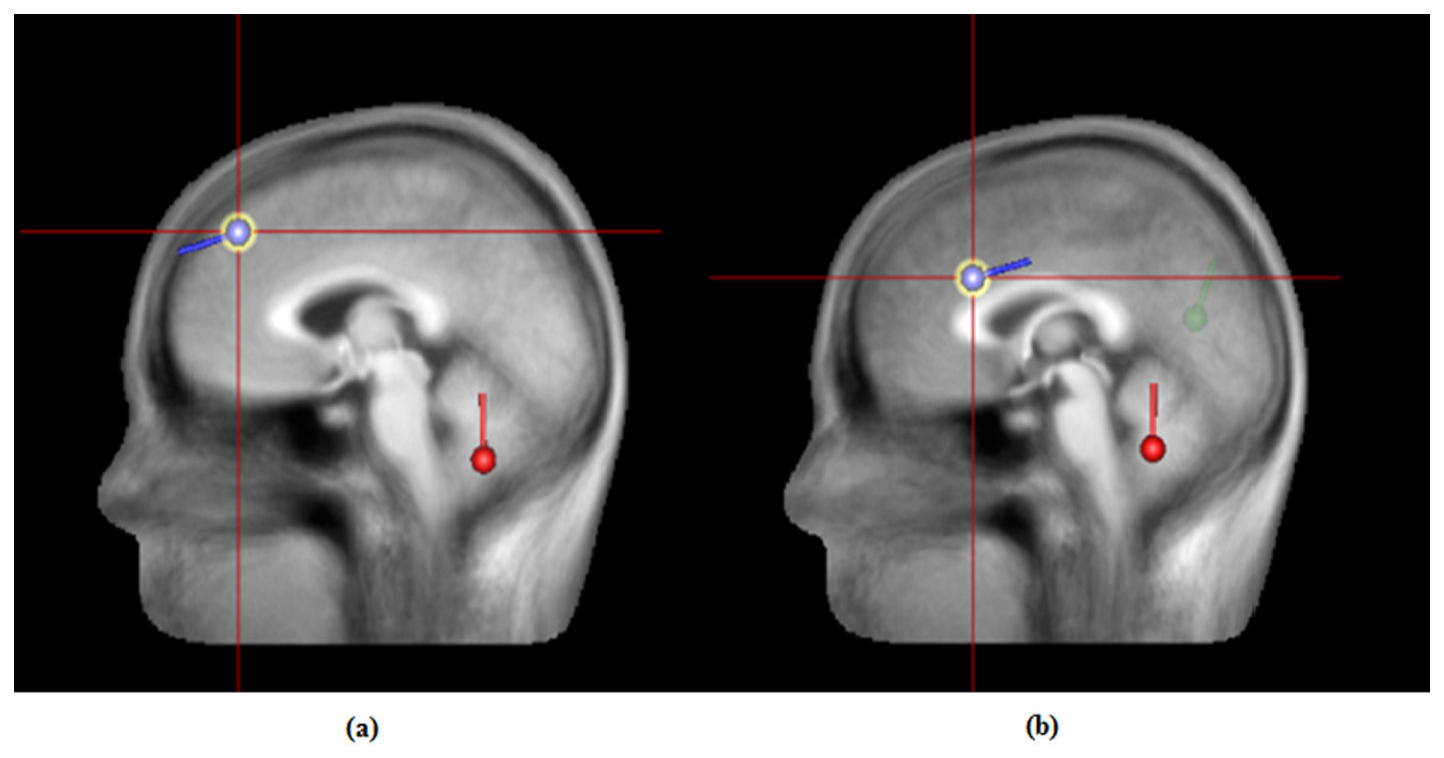
Dipole source localization images of parental faces versus unfamiliar faces for P300 latency. The main different brain regions in response to the father’s and mother’s faces were the right medial frontal gyrus (Figure 6a) and the left anterior cingulate gyrus (Figure 6b); blue spots signify the different regions. Activation of the both of the parental faces was also located in the regions of the cerebellar tonsil (red spot) and the precuneus (green spot).

PCA indicated that three principal components could explain 99.5% of the variance. Therefore, three dipoles in total were fitted with no restriction as to their direction or location. The results indicated that the dipoles were located in approximately the right medial frontal gyrus (Talairach coordinates: *x* = -1.8, *y* = 46.8, *z* = 37.9), the right cerebellar tonsil (Talairach coordinates: *x* = 3.3, *y* = -45.9, *z* = -38.9) and the right precuneus (Talairach coordinates: *x* = 29.0, *y* = -77.0, *z* = 33.6; see Figure 6a). This model best explained the data and accounted for the majority of the variance, with a residual variance of 4.80%.

#### Mother-stranger faces

PCA also indicated that three principal components could explain 99.1% of the variance. Therefore, three dipoles in total were fitted, with no restriction as to their direction or location. The results indicated that the dipoles were located in approximately the left anterior cingulate gyrus (Talairach coordinates: *x* = -7.3, *y* = 39.0, *z* = 16.5), the right cerebellar tonsil (Talairach coordinates: *x* = 1.1, *y* = -45.7, *z* = -41.3) and the right precuneus (Talairach coordinates: *x* = 15.0, *y* = -53.2, *z* = 45.5; see Figure 6b). This model best explained the data and accounted for the majority of the variance, with a residual variance of 6.90%.

Validity was tested through the following steps. First, the display of the residual maps in the time window showed no further dipolar activity; second, no other dipoles could be fitted in the investigated time window by comparing the solution with other plausible alternatives (e.g., bilaterally symmetric dipoles). These tests suggest that the model explained the data in the best manner for the time window.

## Discussion

The present study explored the modulation effects of parental attachment relationships on the neural response to parental faces, integrating the attachment qualities questionnaire (IPPA) and the electrophysiological response to parental faces. Consistent with our hypotheses, the biological significance in parents can be detected by the frontally distributed P3a component. At the facial structural encoding stage indexed by N170, no differences were found with respect to the faces of parents and unfamiliar individuals. However, at approximately 220 ms, both the father’s and mother’s face elicited a more positive N2b over the occipito-temporal region than the faces of unfamiliar individuals. The current study revealed an asymmetric correlation between attachment qualities with parents and the P3a amplitudes evoked by parental faces for the first time. Moreover, within the time window of the P300 family, different brain regions were involved in processing parental faces, which merits further discussion.

### Electrophysiological response to parental faces

With regard to the P3a component of our ERP result, a different temporal pattern emerged in the neural response to parents and unfamiliar individuals, with participants showing a larger mean amplitude toward parental faces. Consistent with the previous finding that the frontal P3a component was only elicited by a personally significant stimulus [5,26], our electrophysiological result indicates that parental faces contain important survival and emotional information and, at the level of a higher cognitive processing stage, individuals’ allocations of resources are split into “salient significance” and “non-relevant”. Moreover, further research is also needed to label the frontal P3a and the novelty P3a [5]. Although both frontal P3a and novelty P3a components were frontally distribution, the frontal-P3a was often obtained in the oddball paradigm that presented two stimuli in a random series. Once the target was salient for humans, the frontal P3a could be elicited; however, the novelty P3a was usually evoked by the novel or unexpected stimulus suddenly appearing in the target-detection oddball paradigm (i.e., as a distracter), relating to the processing of attentional reallocation [30], unusual orientation responses [31] or the stimulus prediction [32]. In contrast, parental faces also elicited more positive P3b components compared to unfamiliar faces. The P3b had a larger amplitude when the categorization decisions were more confident [33] and were linked to the updating of working memory [24,34]. Thus, the enhanced P3b observed in the parietal area for parental faces might indicate greater attentional resource allocation and subsequent retrieval of the stored information related to the parental faces, for correct recognition of one’s own parents. To the best of our knowledge, this study is the first to compare the electrophysiological response to parental faces. No differential neural response to parental faces was found in this study, which suggests that the temporal patterns in the processing of fathers’ and mothers’ faces were similar.

Moreover, the face-specific component N170 that was observed in the current study was not modulated by the face types. As is well known, the N170 is associated with the operation of a neural mechanism tuned to detect human faces [14,35]. This result is a possible indication that the structural encoding stage of faces is not modulated by salient significance and, at the processing stage, individuals merely categorize the stimulus as a “face”. The biographical information behind human faces requires more elaborate processing; some research has provided helpful support for our explanation, for which we found that the N170 is unrelated to the facial familiarity effect [9,17,18]. This finding was also consistent with the model of face processing that proposed that the classification of a face is an essential first step before performing higher cognitive processing toward the faces.

Additionally, both parental faces elicited a smaller N2b potential compared to the faces of unfamiliar individuals. In a recent review, the N2 potential has been divided into three functionally distinct subcomponents, two anterior and one posterior [19]. The larger posterior-temporal N2b was related to the individual and the in-depth processing of a face [20]; for example, a larger N2 was found in a face with a threshold relative to a face with a subthreshold [36], self faces compared with famous faces [37] and famous faces contrasted with unfamiliar faces [38]. The finding of N2b that we observed replicates these studies, with the result that different neural responses were observed between salient and irrelevant stimuli. However, in contrast to previous studies, the larger N2b was observed in unfamiliar faces rather than parents’ faces; one possibility is that because of the familiarity of parents and the requirement for the recognition of parental faces, subjects must promote unique in-depth processing for unfamiliar faces to correctly recognize their parents. These inconsistent tendencies of the N2b merit further investigation in different stimuli modality or task requirements. Furthermore, our findings on N2b are also in line with the two-stage model of face processing proposed by Bruce and Young (1986), which imply that before the processing stage for personal identification or emotional relevance, an early stage of facial perception processing is required.

### Attachment effects on neural correlates associated with parents

Interestingly, we found that the P3a amplitude evoked by the father’s face was positively correlated with the paternal attachment score. The IPPA has three dimensions: trust, communication and alienation. In other words, those college students who experienced their attachment relationship with their father to have more trust and communication and less alienation would be more specifically sensitive to the salient stimuli that are associated with their father. This finding provides support for the suggestion that attachment experiences affect humans’ social brain systems [13].

However, inconsistent with our hypothesis and different from the case of the father, we were surprised that the maternal attachment relationship qualities were not significantly related to the P3a evoked by the stimuli that represent the maternal cue. We suggest that this phenomenon can be supported by evolutionary perspectives that demonstrate that, because of the internal gestation and obligatory postpartum suckling [39], the mother always provides more direct care to their children [40]. Because the infants of many species, including human beings, have their survival dependent more directly on their mothers since birth [41], the absence of attention to maternal cues would likely result in catastrophic circumstances. For example, a human infant who does not adequately pay attention to or efficiently recognize their mother would fail to establish an attachment bond and, worse, would lose the opportunities of satisfying their physiological and psychological needs. Thus, through evolutionary pressure, humans have shown a preference for the maternal face relative to other female faces since the moment that we were born [42,43]. This arrangement probably implies that humans begin life broadly tuned to detect maternal cues, and the special status of maternal faces would narrow the function of the experiences that are needed for progressive specialization. Thus, maternal cues with survival significance, such as a mother’s face, are probably not modulated by the experienced attachment relationship. On the other hand, from the evolutionary perspective, it can also be noted that because of paternal uncertainty, paternal investment to offspring was less than maternal and usually manifested in a more indirect way, with indirect dependence on an individual’s survival [44]. It is possible that the more pronounced a paternal role is, the more salient significance that would be elicited, as indexed by the P3a. Nevertheless, our findings did not imply any advantages or disadvantages between parental faces because the temporal patterns that underlie parental faces were similar. We suggest these two different models of correlation between experienced attachment and the neural correlates of parental faces observed in the current study could be due to the different evolutionary roles of the two parents.

In addition, both the earlier visual components of the N170, the N2b, and the parietally distributed P3b that is evoked by parental faces failed to significantly associate with the attachment relationship. One possible explanation is that because the N170 or N2b observed in the occipito-temporal area likely reflects facial global perception [14,45] and the P3b was more sensitive to the process of target detection [25], rather than containing the social or emotional information of a face, it is plausible that the experienced attachment relationship did not influence these components.

### The neural circuits of parental faces

To further explore the neural correlates that are associated with parental faces, we reconstructed the difference waves between parental faces and unfamiliar faces, within the time window of the P300 family. Our result showed that two different brain regions were involved in the processing of paternal and maternal faces. The activation in response to the father’s face was located in the medial frontal gyrus, while the anterior cingulate gyrus was activated when responding to the face of the mother.

Previous research demonstrated that self-descriptive traits would be better remembered than other-descriptive traits [46]; using such paradigms, neuroimaging researchers found that, compared to other-judgments, enhanced medial prefrontal cortex activation was related to self-judgment [47]. Employing the approach of visual presentation, both one’s own face [48,49] and one’s own body [50] activate the medial frontal gyrus, which suggests that this region is related to self-awareness. With regard to the current study, the results possibly indicate that, for a Chinese individual, the father was also a portion of the self-schema, which extends previous fMRI research that found that, for Chinese individuals, mother-representation overlapped the neural substrate for self-representation, being activated in the region of the medial prefrontal gyrus [51]. Self-representation was sensitive to the cultural background; for East Asians, including Chinese, self appears to be a more interdependent style that emphasizes the fundamental connections between people in social contexts [52]. The self effect observed in the neural correlates of father could be from the impact of the father on Chinese individuals’ daily life.

Alternatively, the dopamine-rich cingulate gyrus has been considered to be an evolutionary specialization of the neocortex [53], having functions that are central to specific processing modules for sensory, motor, cognitive and emotional information [54] and reward-based decision making [55,56]. The P300 was characterized by reflecting the phasic activity of the locus caeruleus-norepinephrine [57]. According to the adaptive gain theory, LC neurons would exhibit two modes of activity, phasic and tonic. Phasic activity of the LC-NE system acts as an attentional filter, inhibiting the neural response to irrelevant stimuli and facilitating the neural response to emotionally or motivational stimuli, whereas tonic activity was observed in a period of task waning [58]. The anterior cingulate cortex plays an important role in evaluating whether the stimuli that we encounter have motivational significance and whether we should act on it, which was suggested to drive directly the phasic activity [58]. Thus, stimuli with salient significance, such as one’s mother, could drive the ACC to enhance the phasic activity of the LC-NE system, to facilitate the attentional resources toward it for further processing. This result is consistent with neuroimaging findings that salient significant stimuli, such as romantic love [59] or fearful emotional expressions that cue dangers for humans [60,61], activated the anterior cingulate cortex.

Additionally, both the precuneus and cerebellar tonsil were involved in the processing of parental faces. Previous research found that the precuneus could be involved in face identity when the faces were familiar or express some level of visual familiarity [49,62,63]. Additionally, the region of the cerebellar tonsil might reflect the processing of top-down attentional control, which was activated when providing participants with cues or when participants correctly responded to inhibition [64]. Several brain regions were involved in the processing of parental faces, which implies that recognizing parental faces is a result of spatially distributed processing that involves multiple areas that play a role in cognitive and social functions. Further investigation into this issue might be necessary.

### Limitations of the current study

Because the neural mechanisms of human cognition are shaped by culture-specific experience [65], our limitations in this study are related to the homogeneous sample of Chinese. Does different culture-specific experience influence the neural correlates of parental cues? For example, would the different social statuses of parents between a patrilineal society and a matriarchal society, which is broadly distributed in the North Indian culture, modulate temporal patterns or functional anatomy that is associated with parental cues? Additionally, considering the different self-concepts between Western and Eastern regions [51], the self-effect that we found in the Chinese sample here also merits further exploration, possibly employing high solution methodology such as fMRI. A better understanding of individuals’ neural correlates of parental stimuli necessitates a sample of diverse cultures and should take cultural background into consideration in the future.

Another limitation relates to the absence of familiar stimuli to control the familiarity effects of parental faces. It might be possible to use other acquaintances as control stimuli; however, this approach does not necessarily solve the issue but instead could add other confounders, such as relationship types or physical proximity [6]. However, it is worth noting that the P3a would not be modulated by the familiarity [5], and the correlation observed between self-reported attachment qualities and ERP patterns in the present study as well as the dipole reconstruction of the P300 response to parental faces also suggests that the neural correlates of parental faces are not merely a familiarity effect.

Objectively, a control task could include parents as the non-target (frequent) faces and unfamiliar faces as the target (infrequent) faces, or to use the faces of both parents and of unfamiliar individuals as target stimuli while a third type of stimulus use as the non-target, could merit further consideration, in order to ascribe the neural response to the selective processing of parents. The findings of studies by Bobes et al., (2007) implied that the processing of a face might contain a binary path: emotional-social information (such as salience) and cognitive operation (familiarity or task requirements), although their findings have strongly proved that the P3b was modulated by the familiarity level rather than the emotional relevance and that the P3a was generated only by salient properties, as well as the P3a findings of Weisman et al., (2011). It is still worthy of further investigation as to whether the current exposure time (frequency) would influence the processing of the parents, not only for the precise ascription of the neural response that underlies parents but also to help to further separate the emotional and cognitive factors that are contained in the P3a and P3b.

## Conclusions

To benefit from the ERP methodology, we combined the attachment qualities and the neural correlates that are associated with parental faces in the present study, offering a first glimpse into the modulation effect of experienced attachment on the neural mechanisms that are involved with respect to human parents. Our study further confirms that the P3a is also sensitive to the biological significance of parents. Furthermore, the salient significance of parents indexed by the P3a potential could be modulated differently by the experienced attachment relationship with parents. The subsequent exploration in the dipole source reconstruction suggested that a father’s face could be related to the self-effect for Chinese individuals; a mother’s face activates the anterior cingulate gyrus for the enhancement of phasic activity in the locus caeruleus-norepinephrine system.

Facial features provide an approach for acquiring important characteristics, such as gender, race, age and emotional state, as well as the personal significance of individuals. To humans, parental faces have more biological significance than merely a familiar face, as used in many research studies. This investigation could be beneficial for obtaining a better understanding of human ethological behaviors, such as kin recognition, and should be of interest to those who study affective faces, attachment relationships, family psychology and evolution.
